# Implementation strategies to promote e-Tracker use in the childhood immunization program in Rwanda – a mixed methods study

**DOI:** 10.3389/frhs.2026.1816682

**Published:** 2026-07-30

**Authors:** Thaoussi Uwera, Ingvild Fossgard Sandøy, J. Frederik Frøen, Eleni Papadopoulou, Hassan Sibomana, Andrew Muhire, David K. Tumusiime, Mahima Venkateswaran

**Affiliations:** 1 Center for Intervention Science in Maternal and Child Health (CISMAC), University of Bergen, Bergen, Norway; 2 Regional Center of Excellence in Biomedical Engineering and e-Health, University of Rwanda, Kigali, Rwanda; 3 Bergen Centre for Ethics and Priority Setting in Health (BCEPS), Department of Global Public Health and Primary Care, University of Bergen, Bergen, Norway; 4 Global Health Cluster, Division for Health Services, Norwegian Institute of Public Health, Oslo, Norway; 5 Maternal, Child and Community Health Division (MCCH), Vaccination programs Unit, Rwanda Biomedical Centre, Kigali, Rwanda; 6 Digitalization Directorate General, Ministry of Health, Kigali, Rwanda

**Keywords:** e-Registry, e-Tracker, facilitation strategy, immunization registry, implementation strategy

## Abstract

**Background:**

In Rwanda, the introduction of a digital immunization registry based in the District Health Information Software 2 (DHIS2), the e-Tracker, has encountered several implementation challenges. This study aimed to design, implement, and evaluate targeted implementation strategies to enhance the adoption and sustained use of the e-Tracker within the National Immunization Program.

**Method:**

We used an implementation research design with a mixed methods approach. We conducted focus group discussions with immunization nurses and co-designed facilitation-based implementation strategies with health workers and implemented them in 12 purposively selected intervention facilities from 48 health facilities that were under-performing on the e-Tracker. The remaining 36 under-performing facilities served as a control group. Evaluations were conducted three months post-implementation with one immunization nurse per facility as respondents, using a process evaluation questionnaire assessing three implementation outcomes: acceptability, appropriateness, and feasibility. We assessed data consistency for the BCG, Penta3 and MR1 vaccines with routine reports before and after implementation of the co-designed strategies.

**Results:**

The co-designed strategies included educational videos, establishment of a collaborative group of immunization health workers to facilitate regular learning, and provision of support from champion users. The intervention group indicated higher acceptability ((Mdn=23 vs. Mdn=20.5, *p* = .026) and feasibility (Mdn=18 vs. Mdn=15, *p* = .002) of using the e-Tracker than the control group. Intervention and control groups had similar scores on appropriateness (Mdn = 22 vs. Mdn=23, *p* = .84). Data consistency for all indicators was nearly 100% in both groups before and after the introduction of implementation strategies.

**Discussion:**

The co-designed strategies improved the acceptability and feasibility of the e-Tracker among immunization health workers, while there was no difference in data consistency. These findings suggest an association between participatory, facilitation-based approaches and nurses’ commitment which in turn may support sustainable use of digital tools.

## Introduction

Good quality immunization data from health systems is essential for monitoring coverage of immunization programs and for decision-making on resource allocation to immunization services. In low- and middle-income countries (LMICs) immunization data is mostly recorded manually using standardized paper forms. The data is then typically summarized on a monthly basis and entered into an electronic database for reporting purposes (1). However, these methods of data recording and summary reporting often have weaknesses such as inaccuracies due to recording errors, incomplete data, over-reporting, and delayed transfer of information to higher levels (2). These issues are particularly prevalent in sub-Sahara Africa, largely because of resource limitations and weak infrastructure (3–7).

To address these limitations in the childhood immunization program, a growing number of LMICs are integrating an Immunization Information System (IIS) and Electronic Immunization Registry (EIR) into their health systems as a structured approach to improve data accuracy, completeness, and timeliness (8). The European Centre for Disease Prevention and Control defines the IIS as “confidential, population-based, computerized information systems that record, store, and provide access to consolidated individual immunization information” (8). The EIRs are part of the IIS, capture vaccination data at the individual level, and allow for monitoring and defaulter and dropout tracking (1, 9) IISs and EIRs can support targeted digital communication with clients (10), such as appointment reminders for defaulters (11). However, implementation challenges persist due to factors such as lack of interoperability with Civil Registration and Vital Statistics (CRVS), costs, lack of fit with current clinical practices, and disruptions to the client-provider interaction (12).

In Rwanda, an EIR known as the e-Tracker was introduced in 2019 and integrated within the District Health Information Software 2 (DHIS2) Tracker platform to digitize the recording and monitoring of individual-level childhood vaccination data (13). Following its initial rollout, the system has been scaled up nationwide. Our findings from an evaluation of the implementation of EIRs in Rwanda in 2021 revealed significant system quality and data quality issues, primarily due to lacking technical functionalities in the e-Tracker such as defaulter tracking, appointment lists and automated reporting, and lack of linkage with Civil Registration and Vital Statistics (CRVS) and national identification agency system (NIDA). Insufficient user training was also identified as a significant gap (14). To maximize its impact, the Ministry of Health issued a directive in October 2022 mandating the complete elimination of paper-based immunization records in favour of exclusive use of the e-Tracker. However, despite steady improvements in data management and system use by health workers, as of 2023, many facilities continued to underutilize the e-Tracker system or maintain parallel paper documentation, limiting the full realization of the benefits of the e-Tracker (15).

Health workers, as the primary end-users, play a critical role in the successful implementation and long-term adoption of EIRs. Co-designed strategies for the uptake of new innovations (in this case the EIR) offer an opportunity to engage health workers meaningfully, especially during the transition period (from paper-based to digital systems) by ensuring that their needs, workflows, and feedback are integrated into the design and deployment process (16). Studies have shown that co-design may improve the success of intervention implementation (17, 18). Our aim was to design and evaluate implementation strategies to support the phased withdrawal of paper-based records and promote more effective use of the e-Tracker by health workers involved in immunizations in primary healthcare clinics in Rwanda.

## Materials and methods

### Study design

**Figure 1:**
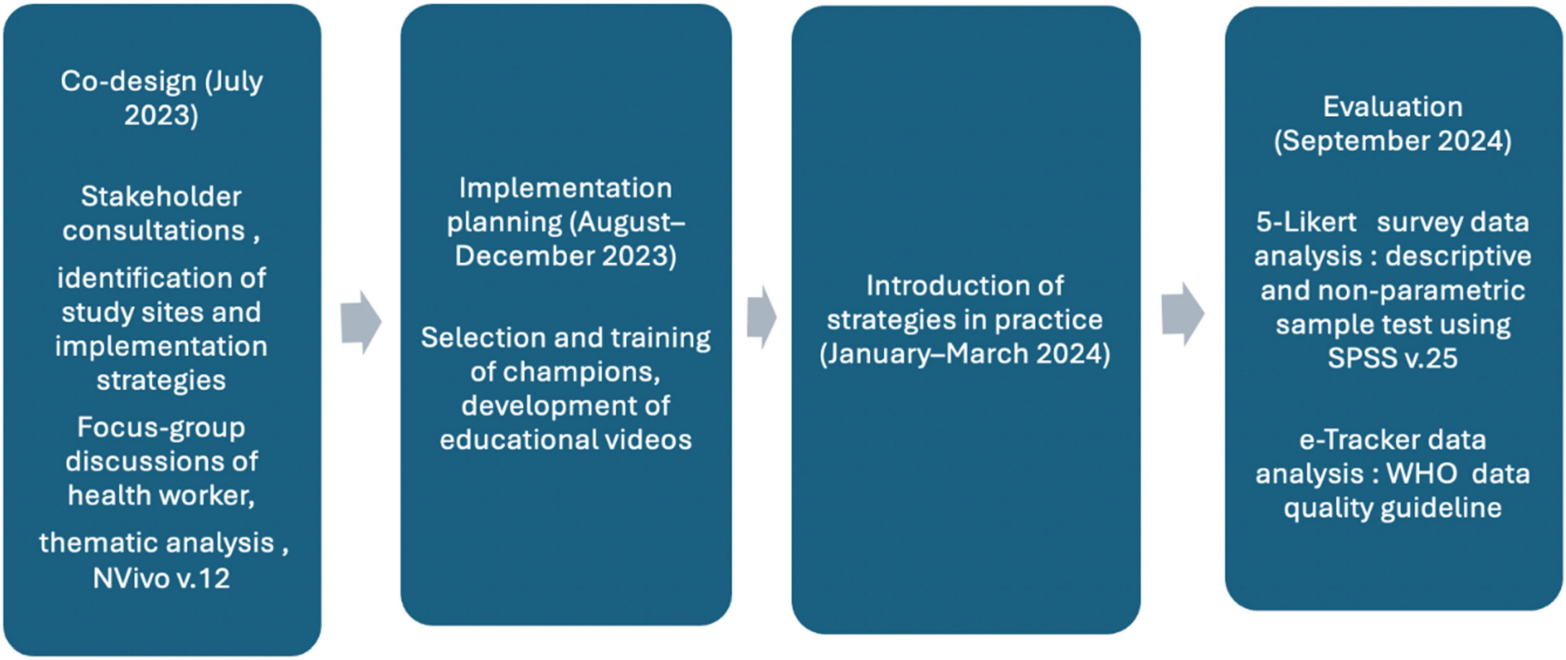
Description of study stages and timeline.

This study employed a quasi-experimental design (19) using a mixed-methods approach and was conducted in two stages in primary health facilities in Rwanda. In the first stage, we organized co-design of tailored, theory-informed implementation strategies. In the second stage, we conducted a process evaluation to assess the implementation strategies. Between July 2023 and September 2024, the sequence of the study was as follows: stakeholder consultations, identification of study sites, focus group discussions, and co-design of implementation strategies (July 2023), planning for the implementation, selection and training of champions, and development of educational videos (August–December 2023), introduction of strategies into practice (January–March 2024), and evaluation of the intervention (September 2024). Figure 1 illustrates the study stages.

### Intervention description

The e-Tracker has been implemented across more than 500 public health facilities. The goal of introducing it was to support real-time data capture, automated reporting, defaulter tracing, and fidelity monitoring at facility, district, and national levels. A phased rollout was conducted between September 2019 and January 2020 and integration with Rwanda's Civil Registration and Vital Statistics (CRVS) and national identification systems took place in early 2022. Health facilities were mandated to transition fully to digital-only immunization documentation by late 2022. Immunization nurses, one per facility, are the primary users of the e-Tracker and are responsible for data entry during immunization sessions. Each health facility also has a data manager.

### Study sample

This study employed a purposive sampling strategy to select health facilities based on their performance in utilizing the e-Tracker system. These performance categories were ranked based on data extracted from the HMIS and e-Tracker reports on all immunization indicators in the period of October- December 2022 by the program managers in the national Expanded Program on Immunization (EPI) at the Rwanda Biomedical Center. The performance assessment entailed examining data consistency of reported immunization indicators across both systems as it reflects the extent to which the digital tool was used correctly, completely and as intended by health facilities (20, 21). Of the total 520 primary health facilities in Rwanda, 472 were classified as “well performing (high data consistency)”, demonstrating 90%–100% consistency between the e-Tracker data and the Health Management Information System (HMIS) reports. The remaining 48 facilities, dispersed across 23 districts, were identified as “less well performing (low data consistency)” with consistency levels below 90% and were all eligible for our study. Of the 48, 12 were in two districts and, for feasibility reasons, these were purposively selected to constitute the intervention group. Two of the intervention facilities were in urban areas (17%) while the other 10 were in rural areas (83%). The remaining 36 facilities (10 in urban areas and 26 in rural areas), scattered across the other 21 districts, served as controls. In this study, the participants were nurses in charge of immunization in the health facilities in intervention and control groups.

### Selection and co-design of implementation strategies

We applied the integrated Promoting Action on Research Implementation in Health Services (i-PARIHS) framework to guide both context mapping and the co-design of implementation strategies (22). We expected that the framework's emphasis on inner and outer context would enable systematic identification of barriers and enablers, and that a focus on recipients and facilitation would support user involvement, and tailored, adaptive implementation assistance, respectively (23, 24). Facilitation here refers to the process of providing support to immunization nurses to use the e-Tracker effectively.

For selecting implementation strategies, we followed methodology aligned with the principles of Implementation Mapping (IM), which emphasize systematically identifying determinants, defining implementation objectives, and selecting strategies whose mechanisms of action address those determinants (25). Given that the system issues of the e-Tracker that were identified in 2021, such as addition of functionalities to support better workflow, and data integration with the Civil Registration and Vital Statistics (CRVS) (14), had already been addressed prior to the co-design, the focus was on facilitation-based strategies that could support effective adoption and sustained use within the health system. Strategies were selected from the ERIC (Expert Recommendations for Implementing Change) framework which categorizes implementation strategies based on their mechanisms of action (26, 27). Specifically, we prioritized ERIC strategies that can support individuals and teams through interactive, context-sensitive methods, namely training, education and mobilizing staff for implementation. The first author (TU) was the external facilitator and led the co-design process and guided the implementation process through planning and executing site-level interactions with internal facilitators who were people within the health system, i.e., health facility leaders, EPI supervisors, and data managers.

The initial set of proposed strategies were presented to national and district-level EPI supervisors and program managers. We subsequently conducted structured engagement sessions with health facility leadership and data managers on the suggested implementation strategies. In addition, two focus group discussions (six participants per group) were conducted with the immunization nurses from the 12 facilities in the intervention group to gather feedback. The first part of the focus group discussions aimed at confirming whether the previously identified barriers to optimal use of the e-Tracker and the discontinuation of paper registers were still relevant; and the second part involved presenting and discussing the proposed list of implementation strategies aimed at overcoming these barriers. The focus group discussions were guided by a structured interview guide (see Supplementary Material 1), developed based on findings from the previous study on barriers to e-Tracker use (14). The final set of strategies were implemented from January 2024.

### Process evaluation

#### Data consistency

Quantitative data on data consistency were retrieved from the national e-Tracker and HMIS databases following a formal request to the Directorate of immunization, Rwanda Biomedical Center. We obtained aggregate data on three indicators: Bacille Calmette-Guérin (BCG), Pentavalent 3 (Penta3) and measles & rubella1 (MR1) vaccines. The three indicators were selected based on the WHO recommendation that a fully immunized child should receive at least one dose of BCG, three doses of pentavalent, and one dose of the measles vaccine (28). The first author (TU) transferred the obtained data into Microsoft Excel and reviewed it for data entry errors before running the analysis. Data consistency was measured between the number of vaccinations reported in the e-Tracker compared to the number of vaccinations reported in the HMIS reports (the former divided by the latter number). This was calculated in both groups per indicator both for the period before (August, September, October 2023) and after (April, May, June 2024) the implementation of the strategies.

#### Feasibility, acceptability, appropriateness

A questionnaire was administered in September 2024 with online links sent via email to the immunization nurses (one per facility) in both groups. The questionnaire included the following measures: the Acceptability of Intervention Measure (AIM), Intervention of Appropriateness Measure (IAM), and Feasibility of Intervention Measure (FIM) as described by Weiner BJ et al. (29), adapted to our intervention. We also evaluated the training since it was the main strategy of the EPI to promote e-Tracker use. Our goal was to assess whether the training received during our study was perceived by immunization nurses as sufficient, had improved their e-Tracker understanding, provided them with sufficient skills to use the e-Tracker, and covered all the challenges faced in the e-Tracker. All the measures used 5-point Likert scales (Supplementary Material 2).

### Analysis

Both focus group discussions were audio-recorded, transcribed, and translated into English by a bilingual professional. The first author (TU) analyzed the transcripts and identified the codes using an inductive thematic approach, while the last author independently verified the codes. All relevant text was coded in line with the study objectives, and emerging themes were discussed with the co-authors. NVivo 12 was used for data management and analysis.

Descriptive analysis was performed to summarize levels of training satisfaction and sociodemographic data such as age, gender, experience, and education. The two groups were compared by using a non-parametric test to assess the differences in implementation outcomes (acceptability, appropriateness, and feasibility of implementation and use of e-Tracker Data) using SPSS software version 25. Due to the small sample size and the non-normal distribution of the data (Cohen's r) is reported to indicate this size of the differences between two groups (30, 31).

Data consistency was analyzed based on the WHO data quality guideline (32), and description of aggregate indicators in HMIS reports versus the e-Tracker are presented.

### Ethical approval

This study was approved by the Rwanda National Ethics Committee (reference: 343/RNEC/2023) and the Regional Ethics Committee for Health Research (REK), Norway (reference: 251925). The participating nurses were informed about the study's objectives and gave their consent before participating.

## Results

### Focus group discussions with immunization nurses

The themes and subthemes from the focus group discussions are summarized in (Table 1). The themes were motivation of nurses to transition to digital only documentation, common barriers to e-Tracker use, and proposed strategies.

**Table 1:** Summary of findings from focus group discussions on barriers and proposed strategies.

Summary of emergent themes and subthemes
Motivation to transition to digital only documentation
Time saved due to removal of double data entry
Safety of client records
Common barriers to e-Tracker use
Connectivity
Difficulties in retrieving defaulters and dropouts in the system
Difficulties in retrieving routine aggregate reports
Implementation strategies for enhanced e-Tracker skills
Educational video content
Collaborative learning champion

Overall, the nurses were positive to transition to digital-only documentations. The main motivations were time saved in avoiding dual documentation on paper registers and the e-Tracker, and data safety. Some of the participants indicated that:

“We are very much happy with that [transition]. In addition, if the system works properly and people use it, this may decrease the time, for instance, one used to spend on paper.” (FGD 2, Nurse 2)

“You realize that where Rwanda is today, they made a good step; …even if something happens the data will not be lost because they are stored safely [in the e-Tracker].” (FGD 1, Nurse 1)

Network connectivity was perceived as the biggest barrier to be able to fully transition to digital documentation. While participants acknowledged that the e-Tracker had become better in terms of technical functionalities, they proposed further technical enhancements to the e-Tracker such as translation of system indicators into the local language, automated pop-up notifications for defaulters, and ability to generate the full immunization history of children who have completed the vaccination schedule.

Participants identified three main challenges with day-to-day use of the e-Tracker, namely, retrieving client records, identification of defaulters, and generating routine reports.

“As for me, what I think you can help us with, is the other way of entering a child, actually that first step, that place where they fill in all the identification of the child. Though we usually fill in that step, it is not very clear. Most of the time you realize that things are even in English. So, you understand that if we get facilitation in Kinyarwanda, this can help us too.” (FGD2, Nurse 5)

“Another thing maybe related to the other way of finding out the dropouts, then you retrieve them and after you have got them, you print the list so that you may see how you can give it to the one in charge of community health workers in order for them to help us find such children.” (FGD2, Nurse 3)

“There is another issue concerning the report. I have not yet given my report today for instance, just because I do not know how to find it and retrieve it. But data manager made it for me, so, I too want to know it so that I may usually do it myself.” (FGD1, Nurse 4)

### Implementation strategies

The national and district-level supervisors, program managers, health facility leadership and data managers were positive to the proposed strategies. Focus group participants were also presented with the proposed facilitation-based implementation strategies, primarily focused on improving individual-level knowledge and skills and with the aim of enhancing e-Tracker use and integrating it into daily routine practice. Nurses suggested videos as a useful resource for them to improve their skills in e-Tracker use, specifically in addressing potential embarrassment related to asking for help.

“With a certain video being there, one can say, how can I make it here and then decide, let me play this video again and see. It can be so helpful to someone who is ashamed to always call his/her colleague [for help].” (FGD 2, Nurse 1)

Peer-to-peer support via WhatsApp was considered as the preferred mode of communication and learning. The participants suggested that exchanges of questions and answers through WhatsApp groups can allow immediate support during immunization session.

“It is a good place on the [WhatsApp] platform. Someone may call you when you are busy! But if he/she posts it on the [WhatsApp] group, while you are busy, someone else can respond. If he/she sends it to the group and asks, “please remind me, how do we do these things?” someone else may be able to immediately support him/her and then the problem gets solved.” (FGD 1, Nurse 4)

Identifying and preparing champions involved equipping individuals who dedicated themselves to supporting and driving implementation (33). In our case, this implied overcoming indifference or resistance to full use of the e-Tracker. The nurses viewed data managers as champion users of the e-Tracker and suggested formalizing the data manager's role as a champion.

“Personally, there are some things I can extract from the [e-Tracker] report, but there are also things I cannot do on my own. Sometimes it needs more advanced knowledge because the system has so many technical details. [This] you can ask the data manager; they are like IT people. They can help you, remind you, and you remind your colleagues.” (FGD 2, Nurse 3)

We made educational materials consisting of six videos aimed at addressing the specific challenges mentioned by the nurses during the co-design. The content included demonstrations of: 1) login and creating profiles for a newborn in the immunization e-Tracker; 2) updating information in client records; 3) adding immunization information for children registered through the Civil Registration and Vital Statistics (CRVS) system; 4) viewing lists of children scheduled for immunization appointments, identify those who missed appointments, and generate printable lists of upcoming and overdue clients; 5) correcting data entry errors; and 6) basics of data analysis, such as reviewing the number of vaccines delivered in the health facility and routine reports for immunization indicators (Table 2). The learning collaborative was operationalized through a WhatsApp group for each district consisting of nurses, data managers and their supervisors since WhatsApp was the preferred mode of communication for the health facility staff. Every two weeks, one educational video was posted in the WhatsApp groups. In addition, the data manager in each of the facilities received training to support nurses (Table 2).

**Table 2:** Implementation strategies (34).

Domain	Strategy: Identifying and preparing champions	Strategy: Educational materials	Strategy: Creating a learning collaborative
Justification	Identifying and engaging champions increase the likelihood of success in programs (33, 55, 56)	Educational materials are fundamental tools for building capacity, and addressing barriers to adoption at individual level (34)	Post-training, on-the-job coaching of immunization nurses is as important as the initial training received (57)
Actors	The director of each health facility, epi supervisors, the external researcher (TU) and an expert e-Tracker facilitator who was supporting e-Tracker implementation.	An expert facilitator from top level e-Tracker implementation team.	One trained data manager (champion) per health facility with excellent knowledge of the e-Tracker, the supervisor in the immunization program, immunization nurses
Actions	Identifying and training of data managers by the expert facilitator so that they can support nurses with e-Tracker use.	Creating recorded instructional videos to guide nurses through challenges in the e-Tracker use.	Participation in discussions in a WhatsApp group for immunization nurses, supervisors and data managers with the goal of supporting each other to address challenges in e-Tracker use
Targets of the action	Create a support structure for the nurses within the health system	Providing easily accessible educational material to support nurses	Immunization nurses using the e-Tracker, to enhance their ability to successfully use the e-Tracker
Temporality	First site visit to the health facility to be conducted by a data manager after a month of remote support	First instructional video shared within a week of establishing the WhatsApp group	The learning collaborative WhatsApp group established within a week of training of champion data managers
Dose	The training for the champion data managers lasted for a full day.	Video released once every two weeks for three months	Members of the group to post questions and concerns anytime. One educational video posted on the leaning collaborative group every two weeks
Implementation outcomes affected	Acceptability, appropriateness and feasibility of e-Tracker use	Acceptability, appropriateness, feasibility of e-Tracker use	Acceptability, appropriateness and feasibility, of e-Tracker use

Our implementation strategies were closely aligned with the principles of facilitation, and operationalized through both internal and external facilitators, consistent with the i-PARIHS framework (34). The external facilitator, in collaboration with an expert facilitator (the e-Tracker trainer from the National immunization directorate in the Rwanda Biomedical Center), provided structured training and ongoing support to the internal facilitators (Table 2). This support focused on both the content of the innovation (the e-Tracker) and the implementation strategies (educational materials, learning collaborative, identifying, and preparing champions). By the end of the two-month period, the internal facilitators were able to independently sustain and champion the implementation strategies within their respective health facilities.

The overall barriers identified, e-Tracker practices before our implementation, the corresponding implementation strategies targeted at the barriers, and the changes in practices observed 3 months after the introduction of the implementation strategies are presented in Table 3.

**Table 3:** Results based on identified barriers and facilitators mapped with the constructs of the i-PARIHS framework of implementation research.

i-PARIHS construct	Barriers mapped in earlier formative assessment July-December 2021	State of e-Tracker system pre-implementation July-December 2023	Implementation strategies introduced to address barriers (*n* = 12 health facilities) January -March 2024	Change in practice 3 months after introduction of implementation strategies April-June 2024
Innovation (e-Tracker)	Poor alignment of e-Tracker with routine workflow and double data entry (both on paper and digitally)	The e-Tracker was used as a secondary data entry tool in some health facilities as immunization data were recorded on paper (immunization facility registers and forms) in immunization sessions and then transferred into the e-Tracker some days later by a data manager or a trained nurse.	Educational materials, learning collaborative group, identifying, and preparing champions.	e-Tracker used as a primary data entry tool at the point of care: immunization data recorded in the e-Tracker by a trained nurse during immunization sessions
Recipient	Limited self-reported knowledge and reported lack of sufficient training in e-Tracker use by immunization nurses	Only one nurse per health facility had received the initial training, perceived to be insufficient by nurses; data typically still handed over in paper forms to data managers to be entered as secondary data in the e-Tracker	Educational materials, learning collaborative group	A pool of additional nurses trained in e-Tracker use to prepare them to step-in to do immunizations in health facilities, if needed
Context	Lack of support from health system managers to facilitate changes at the health facility level	Support remained centralized and was perceived as inaccessible by nurses	Identifying and preparing champions	Health facility leaders and hospital personnel, including EPI supervisors, engaged to support data managers’ championship

EPI, Expanded Programme on Immunization.

### Process evaluation

The three implementation outcome measures acceptability, appropriateness and feasibility were selected to be evaluated in this study because they are commonly used to assess implementation success (29, 35). Drawing on prior implementation research (36, 37), we assessed acceptability by examining implementation stakeholders’ perceptions, particularly immunization nurses, of how agreeable and satisfactory the e-Tracker intervention was. Appropriateness was evaluated in terms of the perceived fit and relevance of the e-Tracker within existing immunization practices, while feasibility assessed the extent to which the e-Tracker could be successfully implemented and used as intended in routine service delivery.

The immunization nurses in all the intervention health facilities (*n* = 12) completed the online questionnaire while nurses from two of the 36 health facilities in the control group did not respond to the questionnaire. The mean age of the participants was 38 years in the intervention group and 39 years in the control group. Generally, most participants held a general nursing qualification with one also having a midwifery background. Overall, participants’ experience ranged from 2 months to 25 years, with a median of 3 years of experience in the intervention group and 2 years of experience in the control group.

A Mann–Whitney U test revealed significant difference between intervention and control group in terms of acceptability (*p* = .026) and feasibility (*p* = .002). The intervention group reported higher scores for both acceptability (Mdn=23) and feasibility (Mdn=18), compared to the control group (Mdn=20.5 and Mdn=15, respectively). The corresponding effect sizes ranged from moderate to large for acceptability (*r* = −.32) and feasibility (*r* = −.45), indicating the practical relevance and perceived usefulness of the intervention (Table 4). Intervention (Mdn=22) and control groups (Mdn=23) had similar scores for appropriateness (*p* = 0.84).

**Table 4:** Results from Mann–Whitney U test.

Measures of intervention	Received intervention?	*N*	SD	Mdn	Mann–Whitney U	*Z*	Asymp. Sig. (2-tailed)	Cohen's *r*
Acceptability	Yes	12	1.7	23	115.5	−2.23	.026	−.32
	No	34	5.21	20.5				
Appropriateness	Yes	12	1.97	22	196	−0.20	.84	−.029
	No	34	5.81	23				
Feasibility	Yes	12	1.47	18	82.5	−3.06	.002	−.45
	No	34	4.36	15				

**Figure 2:**
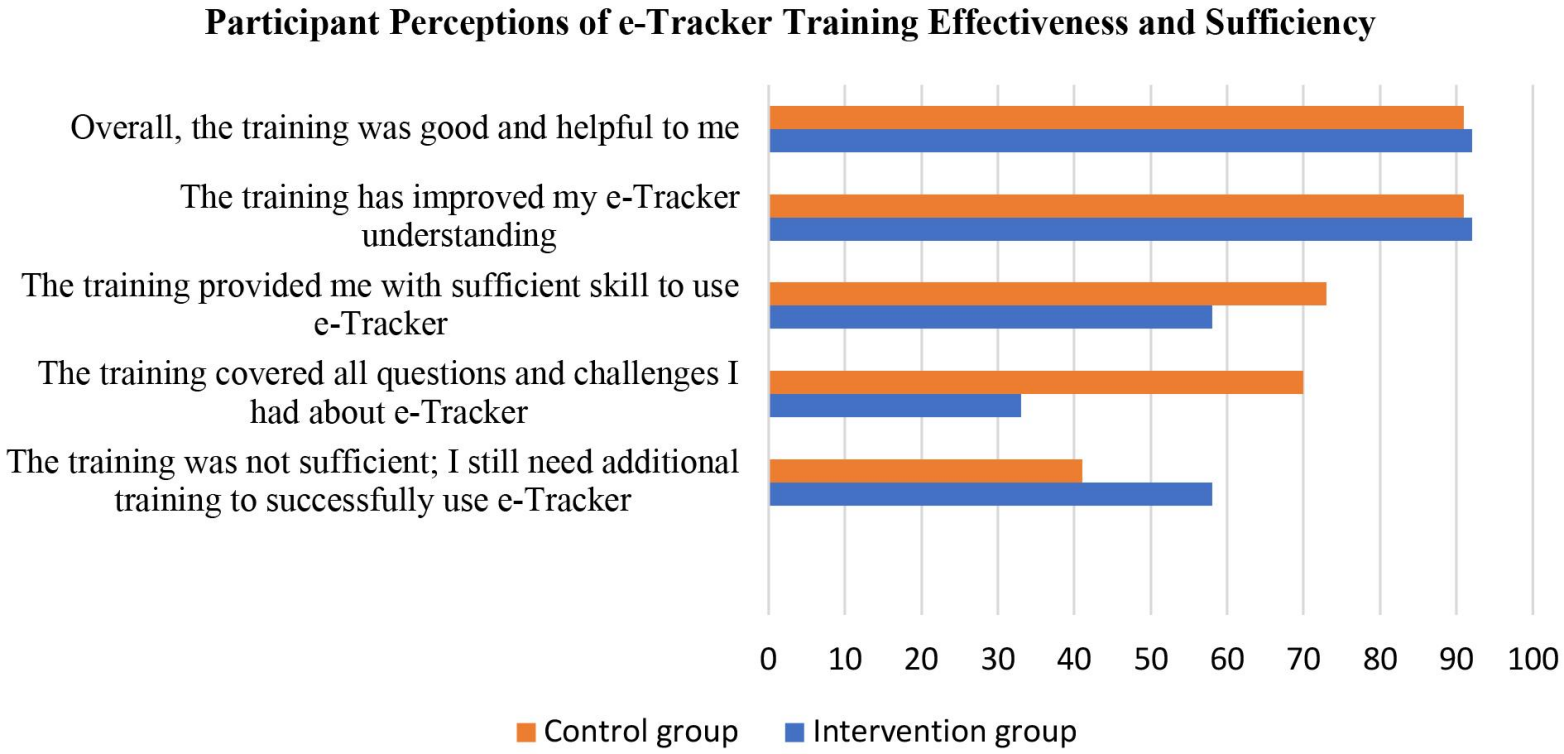
Participant perceptions of e-Tracker training effectiveness and sufficiency.

Between January and March 2024, i.e., during the period of our study, the immunization nurses in health facilities in both the intervention and control groups were offered formal, in-person training sessions from the EPI, in addition to other informal trainings and support from the health system. In the intervention group, 5 out of 12 participants (41%) reported receiving the EPI trainings, compared to 21 out of 34 participants (61%) in the control group. All participants received some form of training during the period. Overall, these trainings were generally perceived as helpful with more than 90% of the nurses who attended indicating that they were useful to improve their understanding of the e-Tracker (Figure 2). In the intervention group, 58% felt the trainings provided them with sufficient skills, compared to 73% in the control group. Only 33% in the intervention group reported that their questions were addressed during the training, while 70% in the control group felt their concerns were covered. Despite these differences, 58% in the intervention and 41% in the control facilities expressed a need for additional training (Figure 2). A similar pattern was observed among those that had received the EPI trainings, with the intervention group reporting lower satisfaction than the control group in terms of content (1 out of 5, 20% vs. 16 out of 21, 76%) and sufficiency (3 out of 5, 60% vs. 19 out of 21, 90%) (Supplementary Material 3, S4).

**Figure 3:**
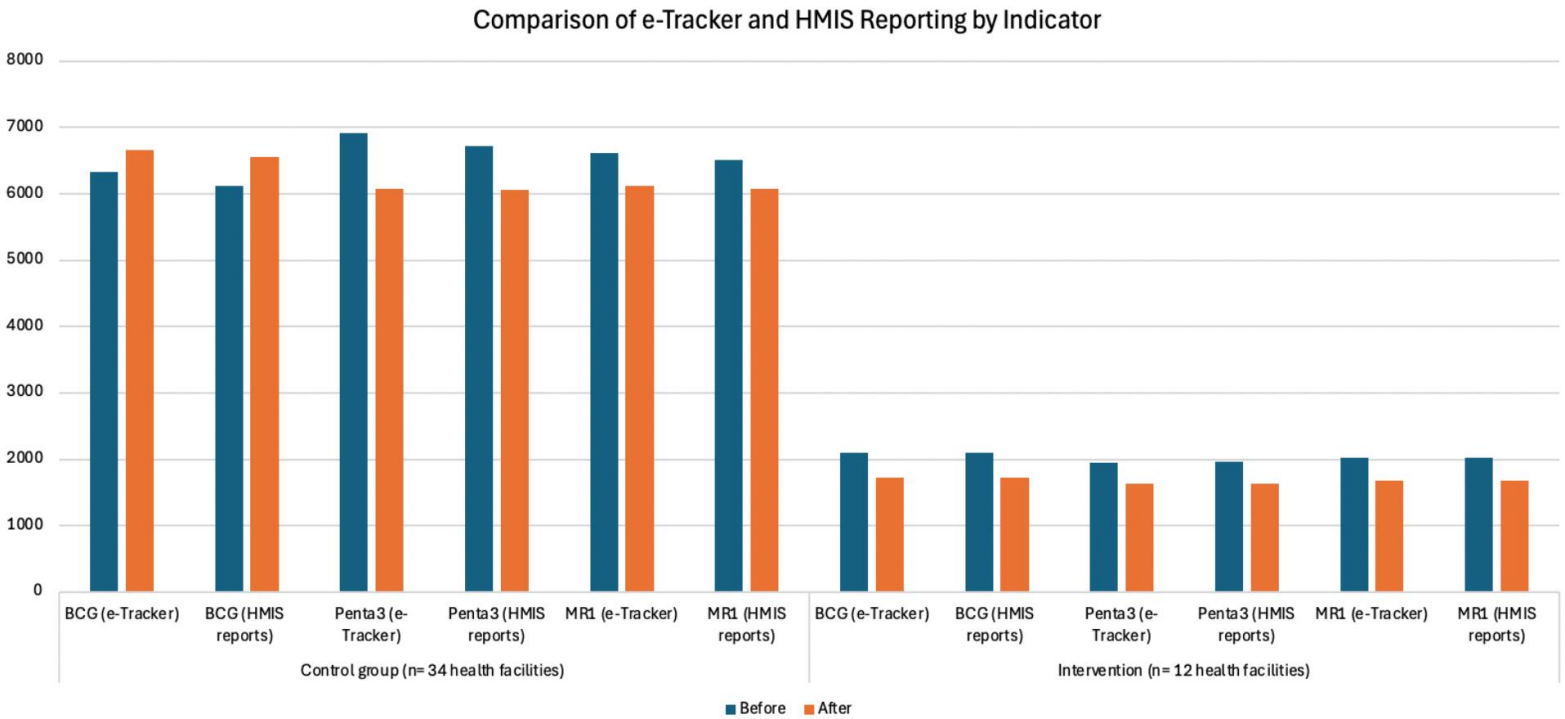
Comparison of e-Tracker and HMIS reporting by indicator before and after intervention (intervention vs. control groups).

**Figure 4:**
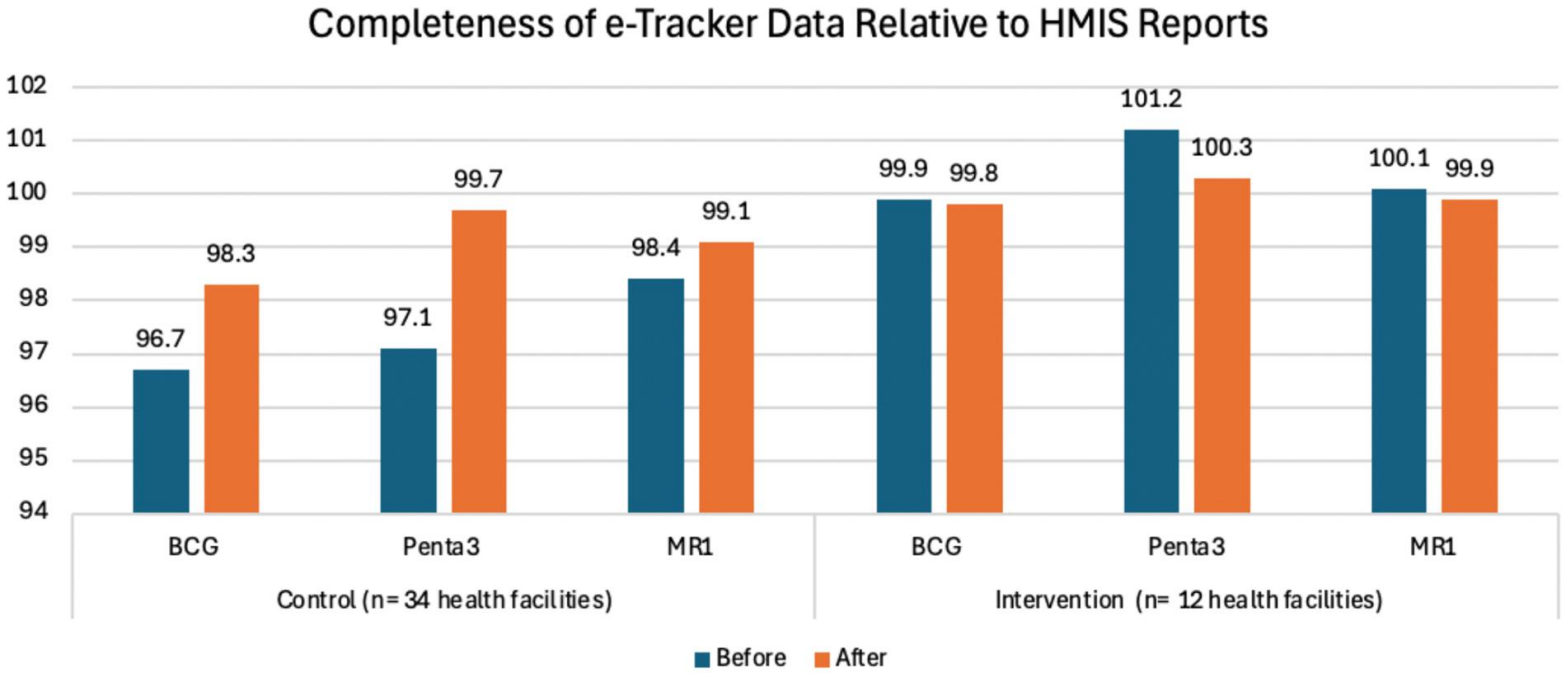
Completeness of e-Tracker data relative to HMIS reports before and after implementation (intervention vs. control groups).

Generally, there was a high data consistency in the e-Tracker use in the months before and after implementation. In the intervention group, recorded BCG, Penta 3 and MR1 data in the e-Tracker and HMIS were closely aligned both before and after implementation of facilitation strategies. In the intervention and control groups, the results indicated minimal differences between recorded e-Tracker data and reported HMIS across all indicators (Figure 3). Before implementation, the data consistency in the intervention group was high, at 99.9% for BCG, at 101.2% for Penta 3, and at 100.1% for MR1. Following implementation, the same indicators continued to show high consistency of 99.8%, 100.3%, and 99.9%, respectively. Similarly in the control group, data consistency was high in the three months before and after implementation: 96.7% and 98.3% for BCG, 97.1% and 99.7% for Penta 3 and 98.4% to 99.1% for MR1 (Figure 4). The detailed month-by-month data for all indicators pre and post implementation for both groups are presented in the tables (Supplementary Material 3).

## Discussion

In this study, we adapted and implemented three facilitation-based strategies to increase the use of the e-Tracker by nurses to record immunization data, namely, educational materials, learning collaborative group, and identifying and preparing champions in 12 health facilities in Rwanda. The findings indicated higher levels of acceptability and feasibility of e-Tracker use in the intervention group compared to the control group whereas perceived appropriateness was similar. Data consistency three months before and after the introduction of the implementation strategies, was similar in the two groups.

Key barriers identified in our previous and current study, such as insufficient training, increased workload due to dual documentation, and limited ongoing support, are commonly reported barriers to the use of digital health systems in other studies according to an umbrella review (38). In similar settings, specifically in sub-Sahara Africa, a realistic synthesis emphasized that even when reliable infrastructure and strong institutional support are in place, digital health technology may often remain underutilized, if users lack confidence in their own ability to use the tools (39). According to the literature, strategies such as those implemented in our study, can alleviate these barriers (34, 38). A study conducted in India showed that on-the-job support enhanced acceptance levels of an eHealth system in primary healthcare (40). Studies done on champions as an implementation strategy have demonstrated higher acceptability of digital health interventions mainly through improved confidence and positive attitudes (33, 41). Gabrielle et al. has demonstrated that collaborative learning groups can reduce feelings of isolation, enhance motivation and acceptability of an intervention (42).

Our evaluation results showed that participants in both groups reported high levels of satisfaction with the training received in terms of quality and helpfulness but also reported that it was insufficient as it did not cover all questions they have had about the e-Tracker. Health workers in several settings in sub-Saharan Africa have highlighted the need for continuous in-house training in the use of digital tools (43). A scoping review of previous studies conducted in similar settings found that although champion-led and digital cascade training approaches improved health workers’ knowledge and acceptance of electronic tools, nurses frequently continued to report insufficient training, limited confidence, and the need for refresher sessions (44).

Studies that compared digital training sessions versus in-person workshops for nurses, revealed that nurses were more satisfied with digital training sessions, perhaps due to the flexibility of digital education considering nurses’ huge workloads (45). However, participants in the intervention group in our study, who received additional support through learning collaboratives and educational materials, did not report increased satisfaction (46). Health facilities in the intervention and control groups continued to receive training through the EPI during the study period. Notably, the control group received this training with slightly greater frequency. The lower score for sufficiency and content of training in the intervention group may be due to an increased expectation of what training sessions should offer. Participants in the intervention group may also have been more accustomed to giving critical feedback to the facilitators, which could have shaped how they responded to questions on training. The small sample size and non-random selection of intervention facilities may have introduced selection bias, with the intervention group potentially including more critical individuals. Social desirability bias may also have influenced control group responses, since they only received trainings by the health system through the EPI (47).

Despite these, the study found high data completeness (ceiling effect) within the e-Tracker, indicating strong data consistency at the operational level. Use of the e-Tracker in Rwanda had increased over time in both intervention and control groups compared to 2022 when we conducted our first assessment (48), with data consistency being nearly 100% in both groups before and after the introduction of implementation strategies. These suggest that, over time the health workers have been increasingly able to enter required information into the e-Tracker system accurately and completely even if they felt inadequately trained, as reported by another study from Rwanda (15). From a user-centered design perspective, facilitation strategies such as additional training, are not only intended to improve immediate technical performance but also to strengthen implementation processes, build user confidence, acceptability, and long-term sustainability (49, 50).

In general, the association between perceptual outcomes, such as acceptability and feasibility, and behavioral outcomes, in this case data consistency, remains poorly understood (51). We did not measure workflow, or the time required for double data entry in the health facilities included in our study. Access to detailed system logs would have enabled a more nuanced understanding of the apparent coexistence of favorable implementation outcomes alongside low satisfaction with the training. Nurses in the intervention arm may have reported higher acceptability and feasibility because the implementation strategies reduced their documentation burden and saved time. In contrast, while double data entry in the control arm did not appear to affect data consistency, a potentially higher workload could explain the lower acceptability and feasibility of e-Tracker use observed in this group.

Our study is one of the few assessing implementation outcomes of digital health interventions such as EIRs in resource-limited settings using implementation science methods. A key strength of this study lies in the collaboration with stakeholders throughout its conduct. Co-design enhances the feasibility of health interventions by ensuring they are contextually relevant, user-friendly, and aligned with stakeholder needs. In Rwanda, initiatives like Cyber-Rwanda and digital mental health programs used participatory design to engage youth, health workers, and policymakers (52), an approach that led to better usability, early identification of barriers, and stronger stakeholder ownership, key factors that support successful implementation and sustainability (53). Similarly in our study, engaging end-users in the design process may have fostered greater ownership and motivation, which may have also contributed to the higher levels of acceptability and feasibility observed in the intervention group.

The study had some limitations. First, it only included 46 health facilities, of which 12 facilities selected from two districts received the implementation strategies based on feasibility and were not randomly assigned to intervention or control. This pragmatic allocation has resulted in an unequal distribution of facilities across study groups and a relatively small number of intervention sites, with a small number of respondents, which may have reduced statistical power to detect modest effects and increased the possibility of Type II error. The number of eligible health facilities with poor data consistency where it was feasible to implement our strategies was pre-determined, hence we did not perform a-priori sample size calculations. Second, contextual factors from baseline imbalances in geographical distribution of the sites may have introduced confounding due to selection bias and have contributed to differences in the assessed outcomes. Furthermore, other parallel interventions, such as training in control health facilities, are likely to have influenced the results. Third, the reliance on self-reported data might introduce the potential for response bias, especially social desirability bias (54).

## Conclusion

Qualitative findings indicated that immunization nurses were positive towards transitioning from a paper-based system to the e-Tracker and valued the planned facilitation strategies. Facilitation-based strategies implemented among immunization nurses in Rwanda was associated with perceived feasibility and acceptability of e-Tracker use. Data consistency, measured by the completeness of e-Tracker data, was high and comparable between intervention and control groups. However, dissatisfaction with the training received suggests that positive e-Tracker use may coexist with perceived gaps in implementation support, that the training delivery may require improvement.

## Data Availability

The original contributions presented in the study are included in the article/Supplementary Material, further inquiries can be directed to the corresponding author.

## Supplementary material

Supplementary Material 1 Focus group discussion guide.

Supplementary Material 2 Process evaluation questionnaire.

Supplementary Material 3 Data for fidelity to the e-Tracker use in the months before and after implementation.
