# Supplementary material for: Implementation strategies to promote e-Tracker use in the childhood immunization program in Rwanda – a mixed methods study
Source: Front Health Serv. 2026 Jul 30;6:1816682. doi: 10.3389/frhs.2026.1816682 (PMC13468503; doi:10.3389/frhs.2026.1816682)
Supplement: Supplementary file 1 [file Datasheet1.pdf]

Focus Group Discussion Guide

Facilitation strategies to improve e-Tracker use for the childhood immunization program in Rwanda

Informed consent

Go through the informed consent form (see separate attachment) with the participants.

General questions

1. How do you perceive aspects of context influencing implementation of e-Tracker?
2. How did you receive ministerial order of phase out papers and keep digital?
    a. Probe: have you been happy with it? why?
    b. Probe: have you been unhappy with it? why?
3. What do you think could be done for you to fully use e-Tracker?
4. The main challenges for you are based on lack of computer skills and e-Tracker training. How do you perceive the following facilitations to make e-Tracker become a routine part of your immunization duties:
    a. Do you think educational materials in terms of recorded videos could improve your skills and knowledge on e-Tracker use?
    b. Do you think creating a collaborative learning group of health workers could improve e-Tracker use?
    c. Do you think creating a group of champions with IT skills, e.g., Data managers to support you can improve e-Tracker use among health workers?
    d. Do you have other suggestions for how you can reduce paper documentations?

Questions on facilitation plan/strategy

5. What do you think about this facilitation plan that we propose implementing to phase out paper documentations?
    a. Probe: is it feasible for you to follow?
    b. Probe: is it possible for you to continue following the plan for a long time?
6. What is the biggest barrier to following the facilitation plan?
    a. Probe: do you get enough support
7. What changes would you make to the facilitation plan?
8. What are your perceptions of the chosen facilitator?
    a. Probe: do you trust the facilitator/facilitators?
    b. Probe: what changes would you make?
