# Supplementary material for: Implementation strategies to promote e-Tracker use in the childhood immunization program in Rwanda – a mixed methods study
Source: Front Health Serv. 2026 Jul 30;6:1816682. doi: 10.3389/frhs.2026.1816682 (PMC13468503; doi:10.3389/frhs.2026.1816682)
Supplement: Supplementary file 2 [file Table1.docx]

Process Evaluation Questionnaire

# Facilitation strategies to improve e-Tracker use for the childhood immunization program in Rwanda

Personal information

1. Study Code:
2. Age:
3. Gender:
4. District:
5. Health center
6. Immunization experience in year at current HC:
7. Educational background:

**Part 1: Measure of intervention**

This Part 1 contains a number of statements about full implementation and use of e-Tracker in health facilities with which some people agree and disagree. Please rate how much you personally agree or disagree with these statements and how much they reflect on how you feel or think personally.

**Acceptability of Intervention Measure (AIM)**

|  | Completely disagree | Disagree | Neither agree nor disagree | Agree | Completely agree |
| --- | --- | --- | --- | --- | --- |
| 1. E-Tracker meets my approval | □ | □ | □ | □ | □ |
| 2. E-Tracker is appealing to me | □ | □ | □ | □ | □ |
| 3. I like the e-Tracker | □ | □ | □ | □ | □ |
| 4. I welcome the e-Tracker | □ | □ | □ | □ | □ |
| 5. E-Tracker is an easy tool to use | □ | □ | □ | □ | □ |
| 6. The layout in the e-Tracker is good | □ | □ | □ | □ | □ |
| 7. E-Tracker increased my documentation burden |  |  |  |  |  |

**Intervention Appropriateness Measure (IAM)**

|  | Completely disagree | Disagree | Neither agree nor disagree | Agree | Completely agree |
| --- | --- | --- | --- | --- | --- |
| 1. E-Tracker seems fitting my work | □ | □ | □ | □ | □ |
| 2. E-Tracker is suitable tool for capturing all child immunization data. | □ | □ | □ | □ | □ |
| 3. E-Tracker seems applicable | □ | □ | □ | □ | □ |
| 4. E-Tracker seems like a good match | □ | □ | □ | □ | □ |
| 5. I can find all the information I need of any immunized child in the e-Tracker. | □ | □ | □ | □ | □ |
| 6. It is difficult to follow-up with children who miss their immunization appointments in the e-Tracker. | □ | □ | □ | □ | □ |
| 7. Reporting immunization data in e-Tracker is easy. | □ | □ | □ | □ | □ |
| 8. E-Tracker is great in making child vaccination history | □ | □ | □ | □ | □ |

**Feasibility of Intervention Measure (FIM)**

|  | Completely disagree | Disagree | Neither agree nor disagree | Agree | Completely agree |
| --- | --- | --- | --- | --- | --- |
|  |  |  |  |  |  |
| 1. All the essentials I need to use e-Tracker are available. | □ | □ | □ | □ | □ |
| 2. The e-Tracker support is available whenever I need it. | □ | □ | □ | □ | □ |
| 3. I have observed the benefits of e-Tracker. | □ | □ | □ | □ | □ |
| 4. I find it possible that I can use the e-Tracker for a long time. | □ | □ | □ | □ | □ |

**Part 2: Measure of facilitation strategies for successful implementation of e-Tracker**

1. Have you ever received any training on e-Tracker? Yes	No
2. Yes	No
3. Yes	No
4. Yes	No

**If no, go to question 2**

**If yes, answer the following questions:**

1.1 When were you trained? January 2024	February 2024	March

5. Yes	No
6. Yes	No
7. Yes	No
8. Yes	No
9. Yes	No
10. Yes	No
11. Yes	No
12. Yes	No
13. Yes	No

1.1.1 Others month specify …………………….

1.2 Was the training about a review of the e-Tracker content that you had previously been trained on (a refresher

training)? Yes	No

14. Yes	No
15. Yes	No
16. Yes	No
17. Yes	No
18. Yes	No
19. Yes	No

1.3 If **no, what the training was about?**

1.3.1. The integration of immunization and nutrition trackers

20. Yes	No
21. Yes	No
22. Yes	No

1.3.2. Get a notification when the next rendezvous is forgotten to be scheduled

23. Yes	No
24. Yes	No
25. Yes	No

1.3.3 Get defaulter pop-up notification after vaccination

26. Yes	No
27. Yes	No
28. Yes	No

1.3.4. Other specify………………………………………………………………………………

………………………………………………………………………………………………………

………………………………………………………………………………………………………

………………………………………………………………………………………………………

………………………………………………………………………………………………………

29. Have you ever watched any videos about e-Tracker use? Yes	No
30. Yes	No
31. Yes	No
32. Yes	No
33. Yes	No
34. Yes	No
35. Yes	No

2.1. If yes, have you ever looked at video again when you have to get quick answer to any problem you

had? Yes	No

36. Yes	No
37. Yes	No
38. Yes	No
39. Yes	No
40. Yes	No
41. Yes	No
42. Has the data manager ever given you support about e-Tracker? Yes	No
43. Yes	No
44. Yes	No
45. Yes	No
46. Yes	No
47. Yes	No
48. Yes	No

3.1 if yes, when have you received a support from a data manager on e-Tracker?

49. Yes	No
50. Yes	No
51. Yes	No

3.1.1 End of last year 2023

h

3.1.2 Beginning of this year 2024

52. Yes	No
53. Yes	No
54. Yes	No

3.1.3 Other time, specify …………………………..

55. Do you have any other people around you who can help you in your work when you have problem with the

e-Tracker? Yes	No

56. Yes	No
57. Yes	No
58. Yes	No
59. Yes	No
60. Yes	No
61. Yes	No

**If answer is Yes, who are they?**

4.1. Epi supervisor

62. Yes	No
63. Yes	No
64. Yes	No

4.2 Others, specify ……………………………………….

65. Do you have a group of people that you can discuss e-Tracker problems with and get answers ? Yes	No
66. Yes	No
67. Yes	No
68. Yes	No

**If No, go to question 6**

**If Yes, answer the following questions:**

5.1 Is this an online group or physical group of people where you meet and discuss?

Online	Physical	Both

5.2. If is online group, when you have trouble with e-Tracker, was it easy to ask questions and receive

answers from the group? Yes	No

69. Yes	No
70. Yes	No
71. Yes	No
72. Yes	No
73. Yes	No
74. Yes	No

5. 3. If is online group, is your group still active? Yes	No

75. Yes	No
76. Yes	No
77. Yes	No
78. Yes	No
79. Yes	No
80. Yes	No
81. In general, how would you rate the training or support you have received on e-Tracker?

6.1 overall, the training was good and helpful to me.

completely disagree	Disagree	neither agree nor disagree	agree	completely agree

82. Yes	No
83. Yes	No
84. Yes	No

v

85. Yes	No
86. Yes	No
87. Yes	No

v

88. Yes	No
89. Yes	No
90. Yes	No

v

91. Yes	No
92. Yes	No
93. Yes	No

v

94. Yes	No
95. Yes	No
96. Yes	No

v

6.2 The training has improved my e-Tracker understanding.

completely disagree	Disagree	neither agree nor disagree	agree	completely agree

97. Yes	No
98. Yes	No
99. Yes	No

v

100. Yes	No
101. Yes	No
102. Yes	No

v

103. Yes	No
104. Yes	No
105. Yes	No

v

106. Yes	No
107. Yes	No
108. Yes	No

v

109. Yes	No
110. Yes	No
111. Yes	No

v

6.3 The training provided me sufficient skill to use e-Tracker.

completely disagree	Disagree	neither agree nor disagree	agree	completely agree

112. Yes	No
113. Yes	No
114. Yes	No

v

115. Yes	No
116. Yes	No
117. Yes	No

v

118. Yes	No
119. Yes	No
120. Yes	No

v

121. Yes	No
122. Yes	No
123. Yes	No

v

124. Yes	No
125. Yes	No
126. Yes	No

v

6.4 The training covered all questions and challenges I had about e-Tracker.

completely disagree	Disagree	neither agree nor disagree	agree	completely agree

127. Yes	No
128. Yes	No
129. Yes	No

v

130. Yes	No
131. Yes	No
132. Yes	No

v vv

133. Yes	No
134. Yes	No
135. Yes	No

v

136. Yes	No
137. Yes	No
138. Yes	No

v

139. Yes	No
140. Yes	No
141. Yes	No

v

6.5 The training was not sufficient; I still need additional training to successfully use e-Tracker.

142. Yes	No
143. Yes	No
144. Yes	No

completely disagree	Disagree	neither agree nor disagree	agree	completely agree

145. Yes	No
146. Yes	No
147. Yes	No
148. Yes	No
149. Yes	No
150. Yes	No
151. Yes	No
152. Yes	No
153. Yes	No
154. Yes	No
155. Yes	No
156. Yes	No

v

157. If you are a new nurse start in your health center, where would you seek help to use the e-Tracker?

7.1 I will seek a special training from the top level

158. Yes	No
159. Yes	No
160. Yes	No

7.2 I will seek continuous support from the trained people around me

161. Yes	No
162. Yes	No
163. Yes	No

7.3 Other specify ………….

164. Have you ever been in a situation where you used other documentation tools than e-Tracker? yes	No
165. Yes	No
166. Yes	No
167. Yes	No
168. Yes	No
169. Yes	No
170. Yes	No
     8.1 If yes, indicate in what situation that happened:

8.1.1 Power issues

171. Yes	No
172. Yes	No
173. Yes	No
     8.1.2 Internet issues
174. Yes	No
175. Yes	No
176. Yes	No
     8.1.3 Device issues (Tablet issues)
177. Yes	No
178. Yes	No
179. Yes	No

8.1.4 E-Tracker issues

180. Yes	No
181. Yes	No
182. Yes	No

8.1.5 Other, specify …….

8.2 In that situation, what have you used to record data?

8.2.1 papers

183. Yes	No
184. Yes	No
185. Yes	No
     8.2.2 Other specify ……………………..
186. From your experience, how far has the transition from paper to e-Tracker come in your health center.

9.1 Papers have completely removed

187. Yes	No
188. Yes	No
189. Yes	No

cv

190. Yes	No
191. Yes	No
192. Yes	No

9.2. We use papers sometimes

9.3 We use papers and e-Tracker on equal match

193. Yes	No
194. Yes	No
195. Yes	No
196. Yes	No
197. Yes	No
198. Yes	No

9.4 We rarely use e-Tracker

9.5 We never use e-Tracker

199. Yes	No
200. Yes	No
201. Yes	No
202. Is there any recommendation you have for how e-Tracker can be more improved? Yes	No
203. Yes	No
204. Yes	No
205. Yes	No
206. Yes	No
207. Yes	No
208. Yes	No

If yes specify: ………………

209. Is there any recommendation you have about training for RBC? Yes	No
210. Yes	No
211. Yes	No
212. Yes	No
213. Yes	No
214. Yes	No
215. Yes	No

If yes specify: ………
