# Supplementary material for: Implementation strategies to promote e-Tracker use in the childhood immunization program in Rwanda – a mixed methods study
Source: Front Health Serv. 2026 Jul 30;6:1816682. doi: 10.3389/frhs.2026.1816682 (PMC13468503; doi:10.3389/frhs.2026.1816682)
Supplement: Supplementary file 3 [file Table2.docx]

**Data for fidelity to the e-Tracker use in the months before and after implementation**

S1 table: e-Tracker data vs. HMIS reports by indicator in intervention group three months before and after implementation of facilitation-based strategies

| Intervention group | | | | | | | | | |
| --- | --- | --- | --- | --- | --- | --- | --- | --- | --- |
|  |  | **e-Tracker** | | | | **HMIS** | | | |
|  | Indicators | Aug | sept | Oct | **Total** | Aug | sept | Oct | Total |
| BEFORE | BCG | 681 | 672 | 752 | **2105** | 678 | 672 | 754 | **2104** |
|  | PENTA3 | 675 | 632 | 638 | **1945** | 699 | 632 | 639 | **1970** |
|  | MR1 | 738 | 655 | 628 | **2021** | 729 | 662 | 634 | **2025** |
|  | Indicators | April | May | June | **Total** | April | May | June | **Total** |
| AFTER | BCG | 625 | 594 | 505 | **1724** | 627 | 589 | 505 | **1721** |
|  | PENTA3 | 548 | 554 | 532 | **1634** | 547 | 556 | 537 | **1640** |
|  | MR1 | 530 | 648 | 498 | **1676** | 527 | 651 | 497 | **1675** |
| Control group | | | | | | | | | |
|  | **e-Tracker** | | | | | **HMIS** | | | |
|  | Indicators | Aug | sept | Oct | **Total** | Aug | sept | Oct | **Total** |
| BEFORE | BCG | 2120 | 1989 | 2226 | **6335** | 2064 | 1856 | 2206 | **6126** |
|  | PENTA3 | 2347 | 2293 | 2278 | **6918** | 2280 | 2228 | 2210 | **6718** |
|  | MR1 | 2261 | 2204 | 2151 | **6616** | 2180 | 2195 | 2138 | **6513** |
|  | Indicators | April | May | June | **Total** | April | May | June | **Total** |
| AFTER | BCG | 2352 | 2347 | 1964 | **6663** | 2312 | 2293 | 1945 | **6550** |
|  | PENTA3 | 1998 | 2166 | 1910 | **6074** | 1980 | 2162 | 1919 | **6061** |
|  | MR1 | 2049 | 2334 | 1739 | **6122** | 2004 | 2327 | 1736 | **6067** |

S2 table: Completeness percentage of e-Tracker (e-Tracker/HMIS reports) before (August -October 2023) and after (April-June 2024) introduction of implementation strategies in the intervention group

|  |  | e-Tracker, n (%) | | | |
| --- | --- | --- | --- | --- | --- |
|  | Indicators | Aug | sept | Oct | Total |
| BEFORE | BCG | 681 (99.5%) | 672 (100%) | 752 (100.2%) | 2105 (99.9%) |
|  | PENTA3 | 675 (103.5%) | 632 (100%) | 638 (100.1%) | 1945 (101.2%) |
|  | MR1 | 738 (98.7%) | 655 (101.0%) | 628 (100.9%) | 2021 (100.1%) |
|  |  | Apr | May | Jun | Total |
| AFTER | BCG | 625 (100.3%) | 594 (99.1%) | 505 (100%) | 1724 (99.8%) |
|  | PENTA3 | 548 (99.8%) | 554 (100.3%) | 532 (100.9%) | 1634 (100.3%) |
|  | MR1 | 530 (99.4%) | 648 (100.4%) | 498 (99.7%) | 1676 (99.9%) |

n=number of records in e-Tracker per indicator

% = HMIS reported number over e-Tracker recorded number

S3 table: Completeness percentage of e-Tracker (e-Tracker/HMIS reports) before (August -October 2023) and after (April-June 2024) introduction of implementation strategies in the control group

|  |  | e-Tracker, n (%) | | | |
| --- | --- | --- | --- | --- | --- |
|  | Indicators | Aug | sept | Oct | Total |
| BEFORE | BCG | 2120 (97.3%) | 1989 (93.3%) | 2226 (99.1%) | 6335 (96.7%) |
|  | PENTA3 | 2347 (97.1%) | 2293 (97.1%) | 2278 (97.0%) | 6918 (97.1%) |
|  | MR1 | 2261 (96.4%) | 2204 (99.5%) | 2151 (99.3%) | 6616 (98.4%) |
|  |  | Apr | May | Jun | Total |
| AFTER | BCG | 2352 (98.0%) | 2347 (97.6%) | 1964 (99.0%) | 6663 (98.3 %) |
|  | PENTA3 | 1998 (99.0%) | 2166 (99.8%) | 1910 (100.4%) | 6074 (99.7 %) |
|  | MR1 | 2049 (98.0%) | 2334 (99.7%) | 1739 (99.8%) | 6122 (99.1%) |

n=number of records in e-Tracker per indicator

% = HMIS reported number over e-Tracker recorded number

| Items | Participants that agree | | | |
| --- | --- | --- | --- | --- |
|  | Intervention group (n=5) | | Control group  (n=21) | |
|  | N | Percent | N | Percent |
| Overall, the training was good and helpful to me | 5 | 100% | 21 | 100% |
| The training has improved my e-Tracker understanding | 5 | 100% | 21 | 100% |
| The training provided me with sufficient skill to use e-Tracker | 3 | 60% | 19 | 90% |
| The training covered all questions and challenges I had about e-Tracker | 1 | 20% | 16 | 76% |
| The training was not sufficient; I still need additional training to successfully use e-Tracker | 3 | 60% | 16 | 76% |

**S4 table: Participants who received EPI training during the study period (Jan, Feb, Mar 2024)**
